# Rnd3 Regulates Lung Cancer Cell Proliferation through Notch Signaling

**DOI:** 10.1371/journal.pone.0111897

**Published:** 2014-11-05

**Authors:** Yongjun Tang, Chengping Hu, Huaping Yang, Liming Cao, Yuanyuan Li, Pengbo Deng, Li Huang

**Affiliations:** Department of Respiratory Medicine, Xiangya Hospital, the Central South University, Changsha, Hunan, P.R. China; Institute of Biomedical Sciences, Taiwan

## Abstract

Rnd3/RhoE is a small Rho GTPase involved in the regulation of different cell behaviors. Dysregulation of Rnd3 has been linked to tumorigenesis and metastasis. Lung cancers are the leading cause of cancer-related death in the West and around the world. The expression of Rnd3 and its ectopic role in non-small cell lung cancer (NSCLC) remain to be explored. Here, we reported that Rnd3 was down-regulated in three NSCLC cell lines: H358, H520 and A549. The down-regulation of Rnd3 led to hyper-activation of Rho Kinase and Notch signaling. The reintroduction of Rnd3 or selective inhibition of Notch signaling, but not Rho Kinase signaling, blocked the proliferation of H358 and H520 cells. Mechanistically, Notch intracellular domain (NICD) protein abundance in H358 cells was regulated by Rnd3-mediated NICD proteasome degradation. Rnd3 regulated H358 and H520 cell proliferation through a Notch1/NICD/Hes1 signaling axis independent of Rho Kinase.

## Introduction

Lung cancer is the leading cause of cancer-related deaths in developed countries. The main types of lung cancer are small cell lung carcinoma (SCLC) and non-small cell lung carcinoma (NSCLC). Despite advances in current medicine, the overall 5-year survival of patients diagnosed with NSCLC is approximately 15%, which indicates that (1) we lack an efficient way to prevent NSCLC; and that (2) we do not completely understand NSCLC. The recurrence of malignancy and chemotherapy resistance are two major limitations in the treatment of lung cancers. Of interest, several malignancies that are resistant to therapy have been closely associated with up-regulated Notch signaling [1]. The role of Notch signaling in NSCLC is controversial. Some studies have reported that active Notch1 inhibited the growth of some NSCLC cells [2], [3], while a more recent study showed that a Notch1 activating mutation in approximately 10% of NSCLC led to a poor prognosis in patients [4]. Therefore, further investigation of Notch signaling in NSCLC is necessary to understand this disease and may be beneficial for the treatment of NSCLC.

Rnd3, also known as RhoE, is a small GTPase involved in the regulation of a wide variety of cell behaviors, including the cytoskeleton, proliferation, migration and apoptosis [5]–[9]. The biological function of Rnd3 was first identified as a ROCK1 repressor that regulates actin dynamics [5]. Recently, studies have reported the broad function of Rnd3 in cancer and neuron system development. Interestingly, Rnd3 is down-regulated in different cancer cells, such as breast cancer cells [8], hepatocellular carcinoma [10], squamous cell carcinoma [11], mesenchymal tumor cells [12], etc. However, studies of the expression and biological function of Rnd3 in NSCLC are very limited.

The role of Rnd3 in regulating the cell cycle was reported a decade ago. Forced overexpression of Rnd3 inhibits cell proliferation and serum-induced S-phase entry, independent of RhoA-ROCK signaling, indicating that complicated signaling is involved in cell cycle regulation [13]. A recent publication explored a very interesting relationship between Rnd3 and Notch signaling [7]. The deletion of Rnd3 resulted in an up-regulation of Notch signaling in mice ependymal cells, promoting proliferation in those cells. RNAi-mediated Rnd3 down-regulation in two cell lines, MDA-MB-231 and HepG2, promoted cell proliferation, cell cycle progression and invasion. However, the role of Rnd3 in lung cancers, specifically in NSCLC, remains unclear. This study, for the first time, uncovers the pathogenic roles of Rnd3 in NSCLC, including the following: 1) Rnd3 is down-regulated in A520, H358 and A549, three lung cancer cell lines; 2) forced expression of Rnd3 in A520 and A358 cells inhibits cell proliferation; 3) the Rnd3-mediated cell proliferation is regulated through Notch signaling regulation via post-translational modification.

## Materials and Methods

### Cell culture and generation of stable cell lines

H358 (ATCC, CRL-5807), H520 (ATCC, HTB-182) and A549 (ATCC, CCL-185) cells were cultured in RPMI-1640 (Life Technologies, Cat# 11875-085) plus 10% fetal bovine serum (Life Technologies, Cat# 16000-044) and 1% penicillin-streptomycin (Life Technologies, Cat# 15140-148). HBEC cells were cultured in Keratinocytes serum-free medium (Life Technologies, Cat# 17005-042) plus bovine pituitary extract (Life Technologies, Cat# 13028-014) and recombinant human EGF (Life Technologies, Cat# PHG0311).

Rnd3 cDNA was subcloned into the lentiviral vector pWPI-polio-eGFP. 293 T (Invitrogen, Carlsbad, CA) cells were transfected with the lentiviral vector expressing Rnd3 and two helper vectors, pMD2 and psPAX2, to produce lentivirus. H358 and H520 cells were then infected with the virus at an MOI of 10. The infection efficiency was verified by GFP expression using fluorescent microscopy. The infected cells were selected by puromycin (2 µg/ml). A single clone was chosen to produce stable cell lines, H358-Rnd3 and H520-Rnd3.

For the cell number measurement experiments, cells were synchronized for 12 h and 10^5^ cells from each group were cultured in growth medium. The cell number was counted every 24 h for a total of 5 days.

### Immunoblotting

Protein samples for western blot analysis were extracted by RIPA buffer (Thermo Scientific, Cat# 89900) plus a protease inhibitor cocktail (Roche, Cat# 11836153001) and phosphatase inhibitors (50 mM NaF, 2 mM Na_3_VO_4_, 4 mM sodium pyrophosphate). Commercially available antibodies were from the following sources: MYPT1 (2634 S), phospho-T853 MYPT1 (4563 S) and PDK1 (3062) from Cell Signaling Technology (MA, USA); MLC2 (PA5-17624) and phospho-Ser20 MLC (PA5-17727) from Thermo Scientific Pierce (IL, USA); ROCK1 (sc-5560) and Rnd3 (sc-53874) from Santa Cruz Biotechnology (CA, USA). Hes1 (ab71559) and Notch1 (ab27526) were from Abcam (MA, USA). Equivalent protein loading was verified by the intensity of a GAPDH (sc20357, Santa Cruz, CA) blot.

### mRNA extraction and qRT-PCR

Total RNA was extracted by Trizol reagent (Life Technologies, 15596-026). qRT-PCR was performed using a SYBR green method with a MasterMix buffer system. The primers sequences were as follows: Rnd3: CTATGACCAGGGGGCAAATA/TCTTCGCTTTGTCCTTTCGT; Jag-1: CTATGATGAGGGGGATGCT/CGTCCATTCAGGCACTGG; Jag-2: TGGGATGCCTGGCACA/CCGGCAGATGCAGGA; Dll-1: GAGGGAGGCCTCGTGGA/AGACCCGAAGTGCCTTTGTA; Dll-4: GCATTGTTTACATTGCATCCTG/GCAAACCCCAGCAAGAGAC; Notch1: CACTGTGGGCGGGTCC/GTTGTATTGGTTCGGCACCAT; Hes1: AGGCGGACATTCTGGAAATG/CGGTACTTCCCCAGCACACTT; GAPDH: GAGTCAACGGATTTGGTCGT/TTGATTTTGGAGGGATCTCG.

### BrdU incorporation assay

Cells were synchronized and then cultured in growth media for 12 h followed by BrdU (bromodeoxyuridine) treatment for 30 min before harvesting. The anti-BrdU antibody (mouse, BD, 552598, 1∶1000) was incubated at 4°C for 16 h, and the secondary goat anti-mouse IgG antibody conjugated with Alexa Fluor 594 (1∶200) was purchased from Invitrogen (A11032). The incubation time was 1 h at room temperature, protected from light. Nuclei were visualized by DAPI (4′,6′diamidino-2-phenylindole) (Vectashield, Vector Laboratories, Inc. H1000), and images were acquired by fluorescence microscopy (DMI 4000B, Leica, Mannheim, Germany).

### Statistical analysis

The data are expressed as means ± S.D. In multiple group comparisons, one-way ANOVA followed by the Student-Newman-Keuls method was used. In 2-group comparisons, an unpaired, two-tailed student’s *t* test was used. All of the analyses were conducted by SigmaPlot 11.0 (Systat, San Jose, CA, USA). A value of P<0.05 was considered statistically significant.

## Results

### Rnd3 is down-regulated along with increased Notch and Rho Kinase activity in H358 and H520 cells

**Figure 1:**
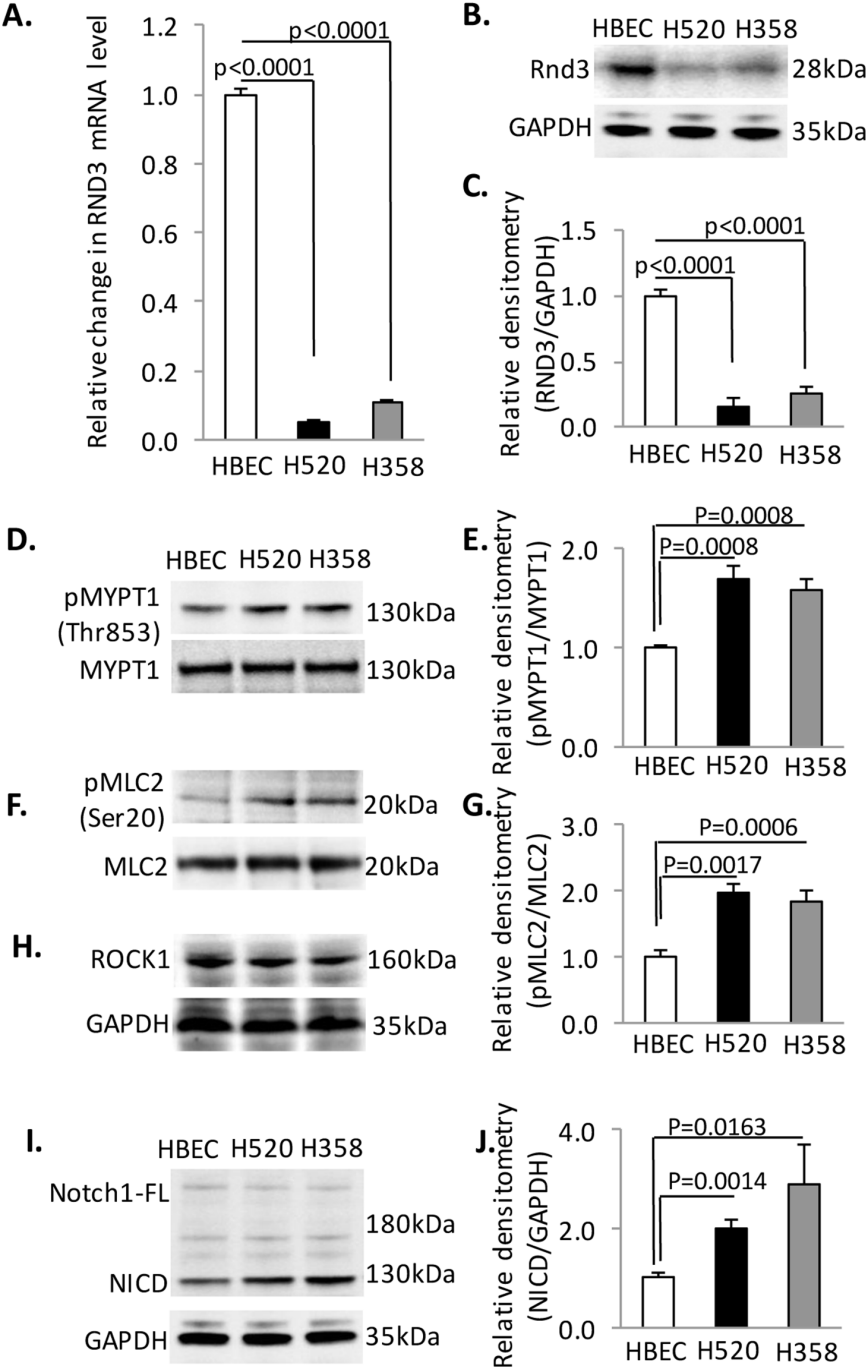
Rnd3 is down-regulated in non-small lung cancer cell lines, H520 and H358. (**A**) Rnd3 mRNA detected by qRT-PCR is down-regulated in H358 and H520 compared to HBEC. (**B**) Rnd3 protein expression levels in cells by western blot. (**C**) Densitometry quantification of western band intensity in B. (**D**), (**F**), (**H**) **&** (**I**) A western blot to detect phosphorylated MYPT1, phosphorylated MLC2, ROCK1 and NICD in cells. (**E**), (**G**) **&** (**J**) Densitometry quantification of western band intensity showed up-regulation of Rho Kinase activity and NICD expression in H358 and H520 cells compared to HBEC. Western blots were quantified from three independent experimental repeats. BrdU-positive cells were quantified from 8 images taken from four slides. Data represent means ± S.D.

Two human NSCLC cell lines, H358 and H520, were used in this study. Human bronchial epithelial cells (HBEC) were used as a normal control. We detected the Rnd3 expression at both the mRNA and protein levels. Compared to HBEC cells, Rnd3 expression was significantly down-regulated in H358 and H520 cells (Fig. 1A–C). Rnd3 was first identified as a ROCK1 endogenous inhibitor that regulates the cytoskeleton. Here, we investigated Rho Kinase signaling by probing two well characterized Rho Kinase substrates in those three cell lines. As shown in Fig. 1D–G, the phosphorylation of myosin phosphatase, target subunit 1 (MYPT1) and myosin light chain 2 (MCL2) was significantly increased in H358 and H520 cells, indicating a hyper-activated Rho Kinase activity. However, the ROCK1 expression level was not different among the cell lines (Fig. 1H). A potent Rho Kinase 1 activator [14], PDK1 which could compete with Rnd3 to bind to ROCK1, expression level was also not changed in the H358 and H520 cells compared to the HEBC cells (Fig. S1). Of interest, in addition to the up-regulated Rho Kinase signaling, we also observed hyper-activated Notch signaling in H520 and H358, compared to HBEC cells (Fig. 1I, 1J), as evidenced by the accumulation of the Notch 1 active form, Notch intracellular domain (NICD). Both Rho Kinase and Notch have been extensively studied in different cells and genetic models. The biological role of activation of these two signaling pathways in NSCLC, and the relationship between Rnd3 down-regulation and NICD up-regulation in lung cancers has not yet been characterized.

### Inhibition of proliferation by stable overexpression of Rnd3 in H520 and H358

**Figure 2:**
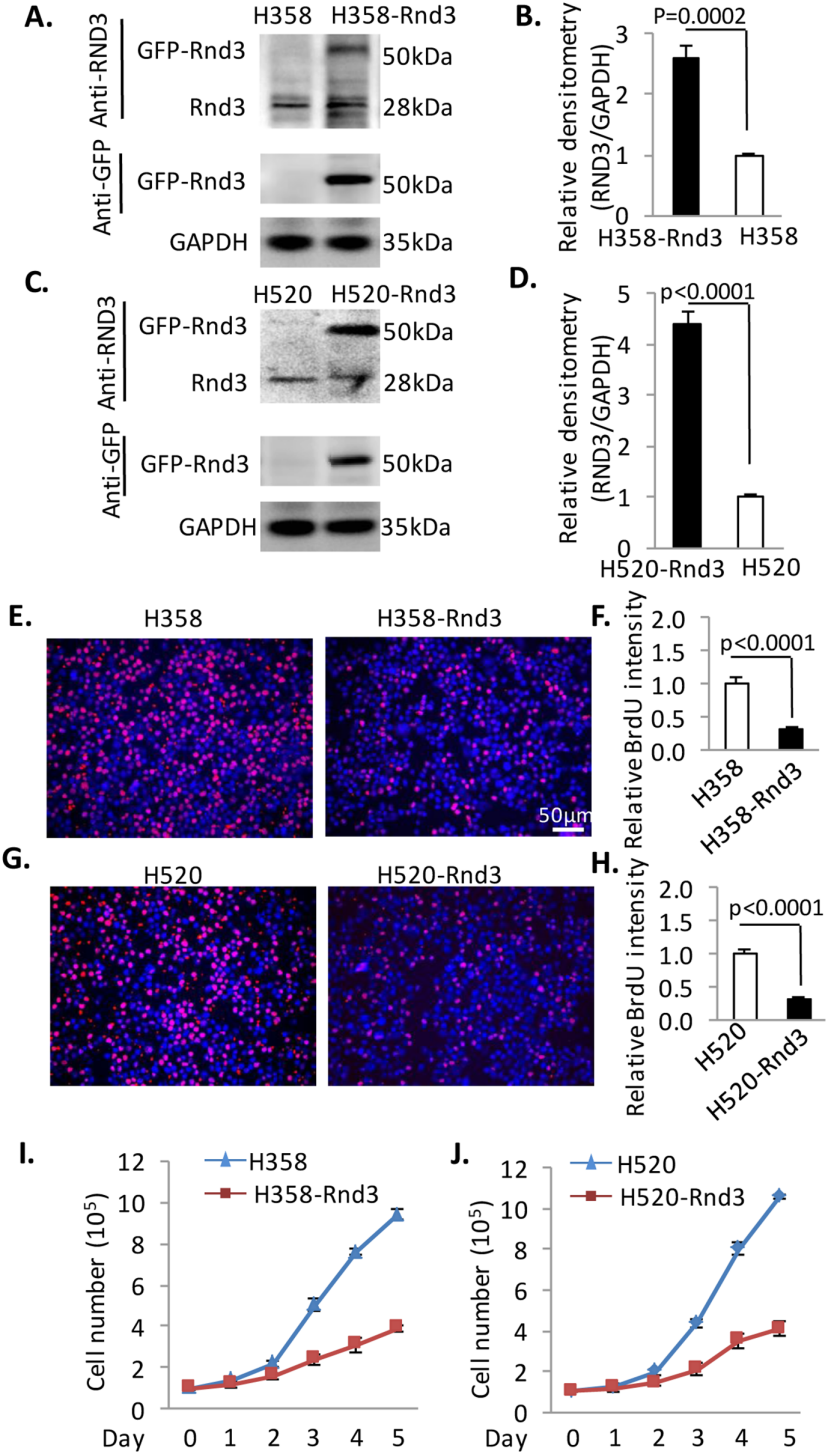
H520-Rnd3 and H358-Rnd3 cells have a lower proliferation rate. (**A**) **&** (**B**) The generation of an H358 cell line stably expressing GFP-tagged Rnd3. The Rnd3 expression was verified by both Rnd3 and GFP antibodies. (**C**) **&** (**D**) The generation of an H358 cell line stably expressing GFP-tagged Rnd3. The Rnd3 expression was verified by both Rnd3 and GFP antibodies. (**E**) **&** (**F**) H358-Rnd3 has a lower proliferation rate compared to H358 cells as detected by BrdU incorporation. (**G**) **&** (**H**) H520-Rnd3 has a lower proliferation rate compared to H520 cells as detected by BrdU incorporation. (**I**) **&** (**J**) Cell number was quantified at different time point. An average of three samples at each time point was presented in this figure. Western blots were quantified from three independent experimental repeats. Data represent means ± S.D.

To investigate the ectopic role of reduced Rnd3 expression in H520 and H358 cells, we generated cell lines stably expressing Rnd3-GFP using a lentivirus system. Rnd3 expression was verified by both anti-Rnd3 (detects endogenous and exogenous Rnd3) and anti-GFP (detects exogenous Rnd3 only) antibodies (Fig. 2A and 2C). H358-Rnd3 and H520-Rnd3 stably expressed Rnd3 at 2.7 times and 4.4 times higher compared to H358 and H520 cells, respectively (Fig. 2B and 2D). Next, we investigated the proliferation rate of the two stable cell lines using a BrdU incorporation assay. The cells were synchronized by serum deprivation and then cultured in growth media for 12 h, followed by BrdU treatment for 30 min before harvesting. The cells were transferred to slides by cytospin for staining and imaging. The proliferation rate indicated by BrdU positive staining was significantly decreased in H358-Rnd3 cells compared to H358 cells (Fig. 2E and 2F). The same result was observed in H520-Rnd3 cells compared to H520 cells (Fig. 2G and 2H). We also counted the ratio of BrdU+ cell to GFP+ cells as shown in Fig. S2. The ratio was significant higher in H358 (50%) and H520 (41%) cells compared to H358-Rnd3 (15%) and H520-Rnd3 (11%) cells, respectively (Fig. S2A and S2B). Furthermore, 1×10^5^ cells from each group were synchronized and then cultured. The cell number was counted at different time points (Day 0, 1, 2, 3, 4 and 5). We observed a significant increase in cell number in H358 and H520 cells. However, this increase was remarkably attenuated in both H358-Rnd3 and H520-Rnd3 cells started at Day 2 (Fig. 2I and 2J). Taken together, forced overexpression of Rnd3 in H358 and H520 could reduce the proliferation rate of these cells. Inhibition of tumor cell growth is one of most important strategies in treating cancer. The role of Rnd3 in inhibiting the proliferation of these two NSCLCs makes it a possible target for cancer treatment.

### Reintroducing Rnd3 blunted Rho Kinase and Notch signaling in H520 and H358 cells

**Figure 3:**
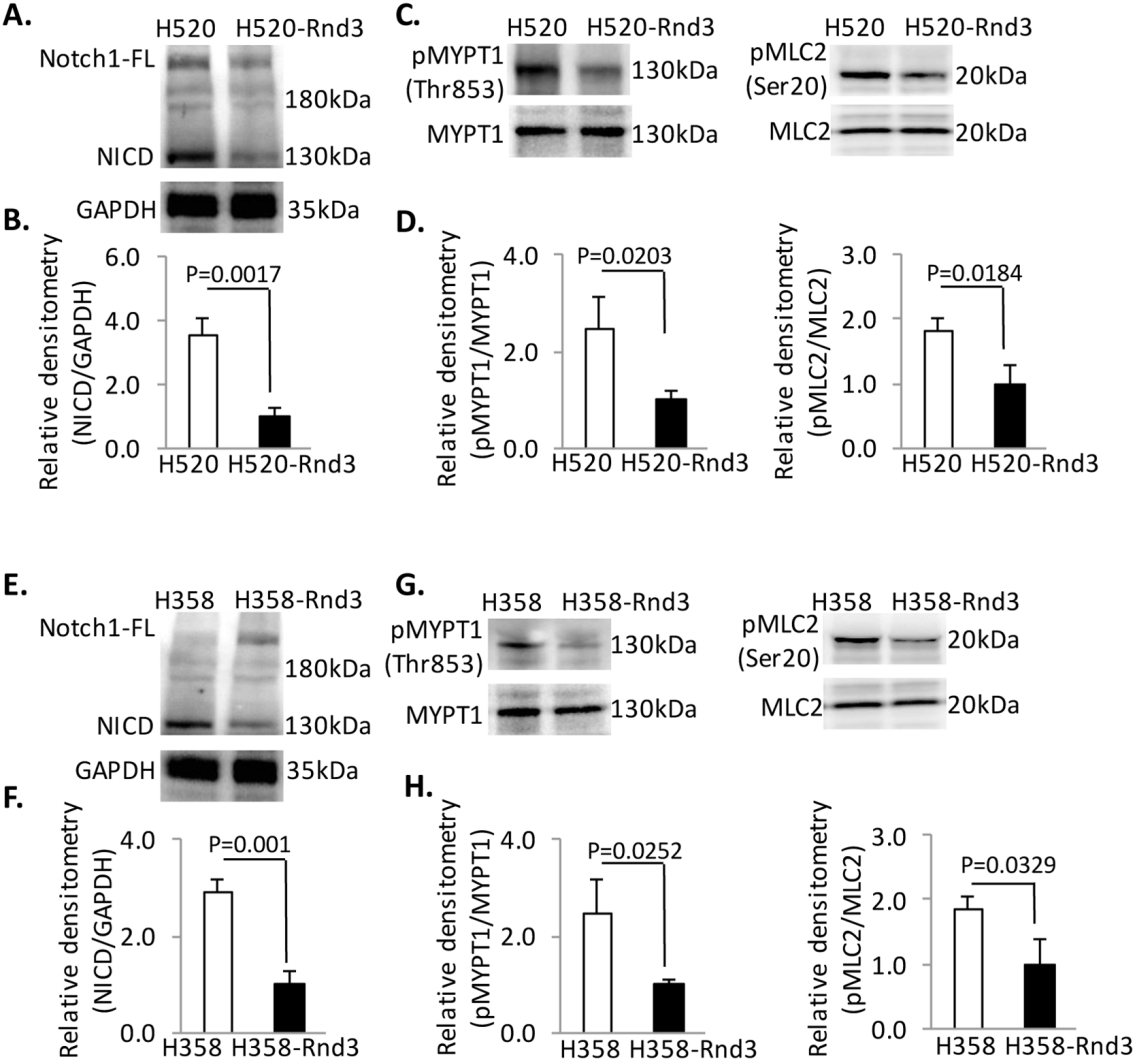
Reintroduction of Rnd3 corrects Rho and Notch signaling in H520 and H358 cells. (**A**) **&** (**B**) Decreased NICD expression in H520-Rnd3 cells compared to H520 cells. (**C**) **&** (**D**) Decreased Rho Kinase activity in H520-Rnd3 cells compared to H520 cells detected by antibodies specific for pMYPT1 and pMLC2. (**E**) **&** (**F**) Decreased NICD expression in H358-Rnd3 cells compared to H358 cells. (**G**) **&** (**H**) Decreased Rho Kinase activity in H358-Rnd3 cells compared to H358 cells detected by antibodies specific for pMYPT1 and pMLC2. Western blots were quantified from three independent experimental repeats. Data represent means ± S.D.

We showed higher Rho Kinase activities and NICD expression together with a reduced Rnd3 expression level in H520 and H358 cells compared to the normal lung epithelial cells, HBEC (Fig. 1). We next explored whether a correlation exists between lower Rnd3 expression and higher Rho Kinase and NICD expression. The NICD expression was down-regulated more than 3.5-fold in H520-Rnd3 cells compared to H520 cells (Fig. 3A and 3B). The phosphorylated myosin light chain phosphatase, subunit 1 (pMYPT1) and myosin light chain (pMLC2) were down-regulated 2.5- and 1.8-fold, respectively, in H520-Rnd3 cells compared to H520 cells (Fig. 3C and 3D). In the H358 cells, we observed the same trend of NICD expression and Rho Kinase activity when Rnd3 was overexpressed. The NICD expression was decreased 2.9-fold in H358-Rnd3 cells compared to H358 cells (Fig. 3E and 3F), while pMYPT1 (2.46-fold) and pMLC2 (1.83-fold) were also decreased in H358-Rnd3 cells compared to H358 cells (Fig. 3G and 3H). Furthermore, overexpression of Rnd3 in H358 and H520 cells did not change the expression of Notch ligand as shown in Fig. S3. Expression of human Jagged-1 (Jag-1), Jagged-2, (Jag-2), Delta-like-1 (Dll-1) and Delta-like-4 (Dll-4) mRNA were measured by qRT-PCR. There is no statistical significant change in expression of those mRNA in H358 and H520 compared the H358-Rnd3 and H520-Rnd3, respectively (Fig. S3A and S3B). These outcomes suggest that Rnd3 could regulate both Rho Kinase and NICD signaling in H358 and H520 cells. Rnd3 is a regulator inhibiting Rho Kinase activation and NICD expression in NSCLCs. Rnd3 mediated Notch signaling modulation did not through regulating the Notch ligand expression.

### Inhibition of Notch signaling prevented proliferation of H520 and H358 cells

**Figure 4:**
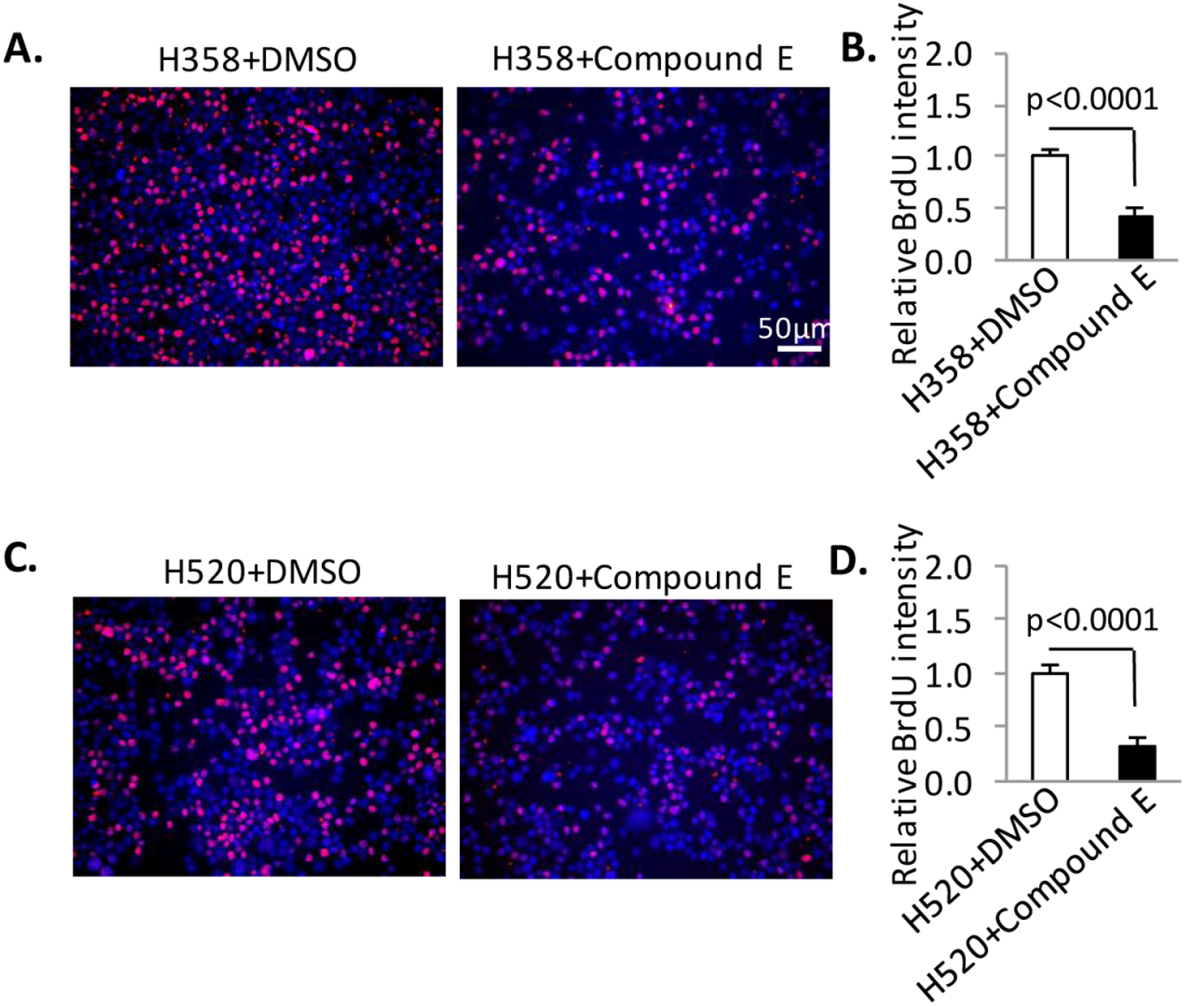
Inhibition of Notch signaling prevented proliferation of H520 and H358 cells. (**A**) **&** (**B**) The application of compound E blocks the proliferation of H358 cells as detected by a BrdU incorporation assay. The cells were treated with compound E at the final concentration of 5 nM and synchronized by serum depletion followed by growth in media for 12 hours. Then, the cells were treated with BrdU for 30 minutes before being harvested for analysis. (**C**) **&** (**D**) The application of compound E blocks the proliferation of H520 cells as detected by a BrdU incorporation assay. The cells were treated with compound E at the final concentration of 5 nM and synchronized by serum depletion followed by growth in media for 12 hours. Then, the cells were treated with BrdU for 30 minutes before being harvested for analysis. BrdU-positive cells were quantified from 8 images taken from four slides. Data represent means ± S.D.

We demonstrated that forced overexpression of Rnd3 by lentivirus in both H520 and H358 cells 1) inhibited proliferation (Fig. 2) and 2) blocked the activation of Rho Kinase and Notch/NICD signaling. To determine which signaling pathway may contribute to the proliferation rate, chemical inhibitors were applied to the cells. Whether Fasudil, a Rho Kinase inhibitor [15], [16], can affect cell proliferation rate in H358 and H520 cells? Our data showed that inhibition of Rho Kinase activity by Fasudil did not alter the proliferation of H520 and H358 cells (Fig. S4). However, compound E [17], a specific Notch signaling inhibitor, can attenuate proliferation in both cell lines (Fig. 4). Treatment with compound E reduced the proliferation of H358 by 40% compared to the DSMO treated control group as indicated by a BrdU incorporation assay (Fig. 4A and 4B). The proliferation rate decreased to only 32% of the control in the H520 group (Fig. 4C and 4D). To test the possible cytotoxicity of Compound E, we evaluated the cell death by Trypan Blue staining. At the concentration of 5 nM compound E, we did not detect a significant cell death compared to non-treatment group in both H358 and H520 cells (Fig. S6, picture 2 and 6 compared to 1 and 5, respectively), suggesting the cytotoxicity of Compound E in all the three cell lines at this concentration was minimal. We further increased the compound E concentration to 50 nM and 500 nM, and found that only less than 5% cell death was observed in 50 nM group (Fig. S6, picture 3, 7 and 11), while massive cell death (>50%) was caused by high dosage compound E (500 nM, Fig. S6, picture 4, 8 and 12). The experiment using selective signaling inhibitors suggested that Rnd3 down-regulation mediated NICD up-regulation may play a more important role in the proliferation of H358 and H520 cells and that this proliferation is not Rho Kinase dependent.

### Rnd3 regulates proliferation by modulating the NICD/Hes1 signaling axis in H520 and H358 cells

**Figure 5:**
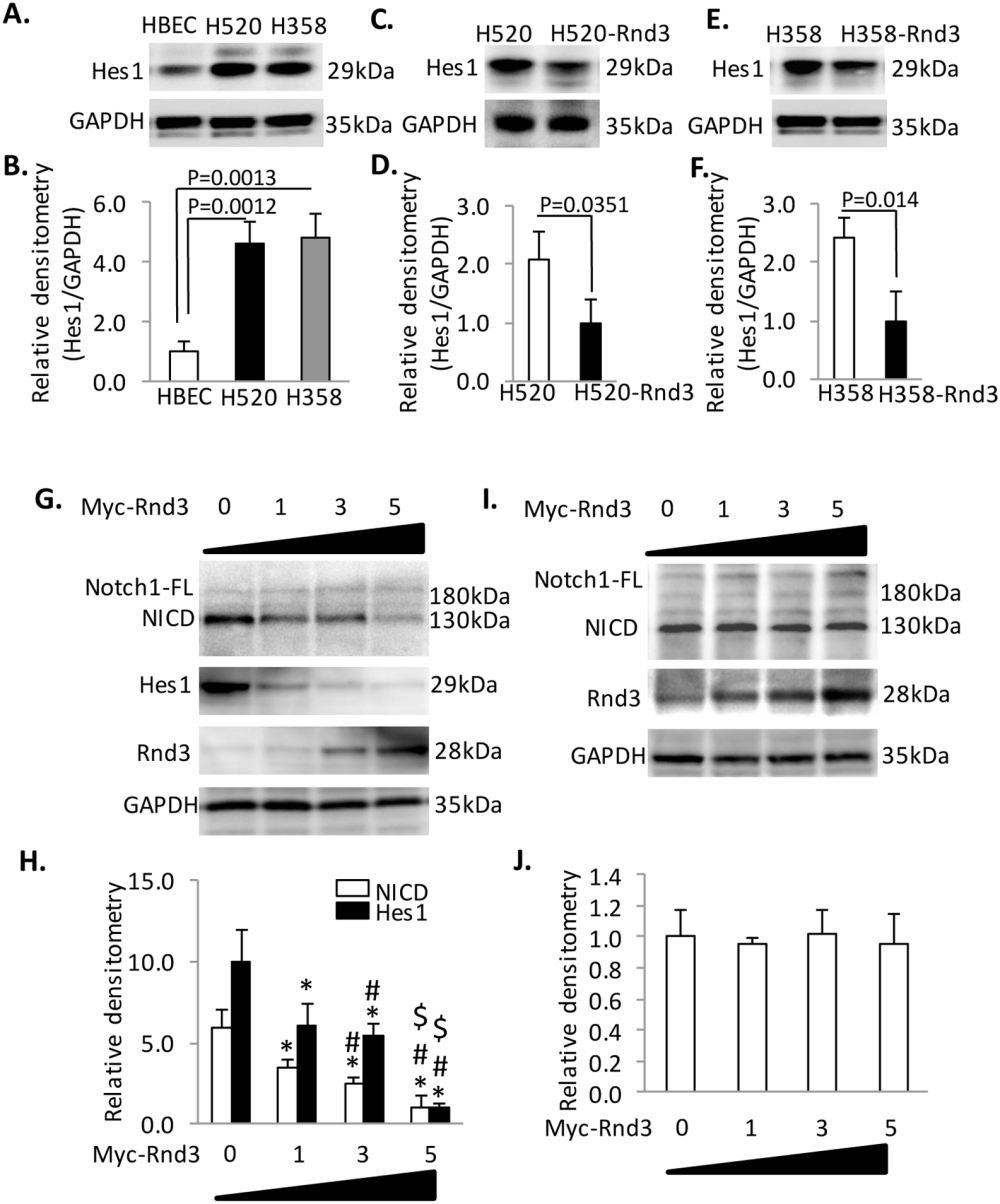
Rnd3 inhibits proliferation by promoting NICD degradation in H520 and H358 cells. (**A**) **&** (**B**) Hes1 was up-regulated in H520 and H358 cells compared to HBEC cells. (**C–F**) Hes1 expression decreased when Rnd3 was stably overexpressed in H358 and H520 cells. (**G**) **&** (**H**) Transient overexpression of Rnd3 in H358 cells down-regulated NICD and Hes1 in a dosage dependent manner. (**I**) **&** (**J**) Inhibition of proteasome activity by MG132 (final concentration of 15 µM) abolished the Rnd3 dosage dependent NICD down-regulation in H358 cells. Western blots were quantified from three independent experimental repeats. Data represent means ± S.D. *p<0.05 compared to control (group 0); **#** *p*<0.05 compared to group 1; **$** *p*<0.05 compared to group 3. Data represent means ± S.D.

Notch signaling is an important cell cycle regulator. Blocking Notch signaling inhibits the growth of different cells [18]–[20]. Noticeably, a recent study by Chang et al. group showed that NICD regulates epidermal cell proliferation through up-regulation of Hes1 in Rnd3 knockout mice [21]. Therefore, we investigated Hes1 expression in our cells. Hes1 was up-regulated 5-fold in H520 and H358 compared to the HBEC cells (Fig. 5A and 5B). Consistent with an attenuated proliferation rate observed in H520-Rnd3 and H358-Rnd3 cells (Fig. 3E–3H), Hes1 expression was also down-regulated by 2-fold and 2.5-fold in these two cells lines compared to H520 and H358, respectively (Fig. 5C–5F), suggesting that NICD inhibition-mediated decreases in the proliferation rates of H520-Rnd3 and H358-Rnd3 cells may be through down-regulated Hes1 expression.

To define the relationship between Rnd3 and NICD, we performed a dosage dependent expression of Rnd3 in H358 cells. myc-Rnd3 was transiently transfected into H358 cells at different dosages (0, 1 µg, 3 µg, 5 µg). Then, we examined the expression of NICD and Hes1 by western blot. With increased Rnd3 expression, the NICD and Hes1 expression levels decreased (Fig. 5G and 5H). Not surprisingly, Hes1 mRNA, an NICD target gene, changed accordingly (Fig. S5 filled black bar). However, we did not observe any change in Notch1 mRNA (Fig. S5 empty bar), despite the significant decrease in protein level due to Rnd3 overexpression, suggesting that Rnd3 regulated NICD at the post-translational level, because the NICD level is regulated by proteasome degradation [22]. We inhibited proteasome activity using MG132 and found that the Rnd3 dosage dependent NICD degradation was completely blocked (Fig. 5I and 5J), suggesting a post-translational regulatory role of Rnd3 in NICD degradation.

### Rnd3 regulates proliferation through Notch1 signaling in lung adenocarcinoma cells

**Figure 6:**
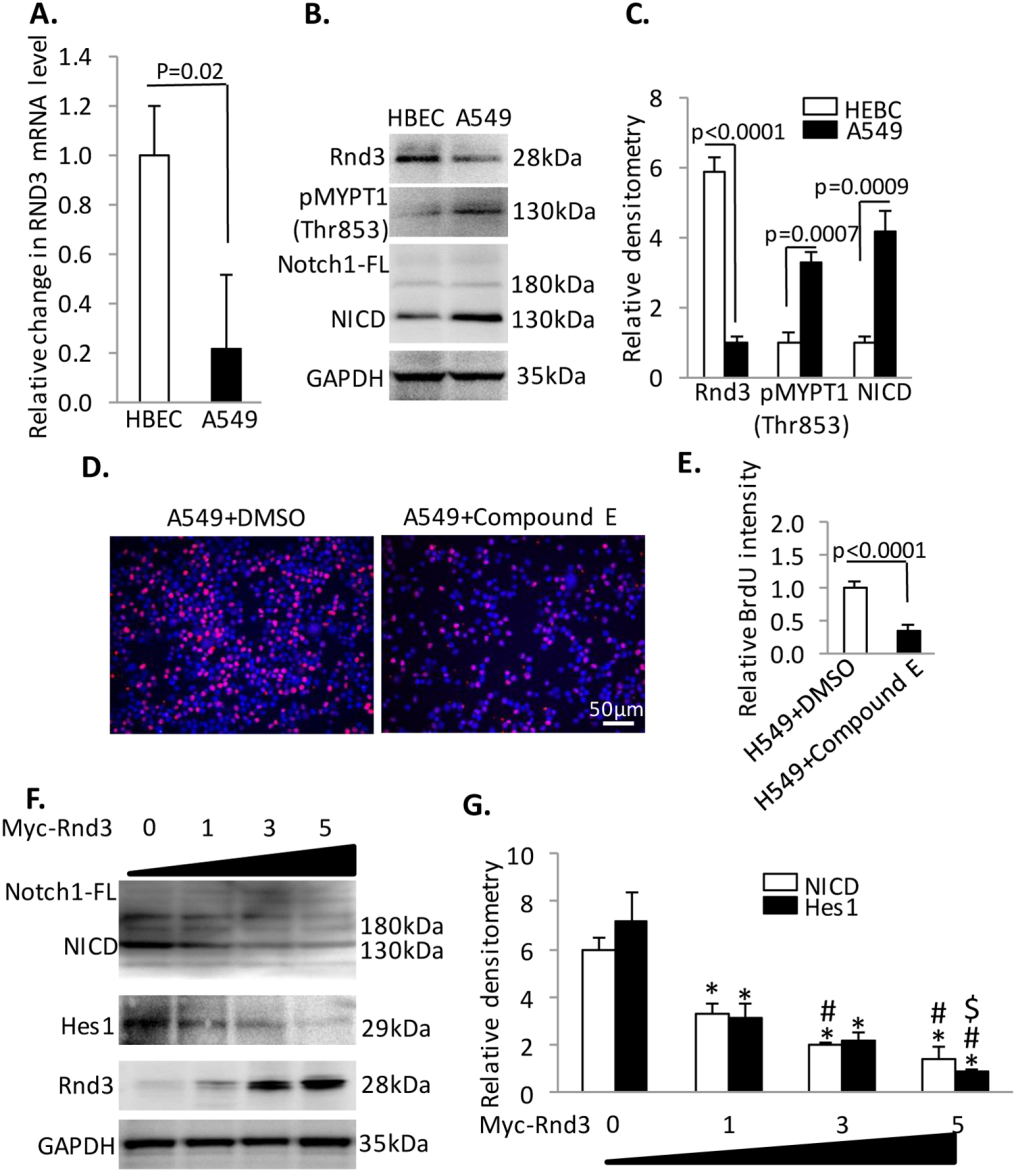
Rnd3 regulates cell proliferation through NICD signaling in lung adenocarcinoma cells, A549. (**A**) Rnd3 mRNA detected by qRT-PCR is down-regulated in A459 compared to HBEC. (**B**) **&** (**C**) A western blot to detect phosphorylated MYPT1, NICD in cells. (**D**) **&** (**E**) The application of compound E blocks the proliferation of A459 cells as detected by a BrdU incorporation assay. The cells were treated with compound E at the final concentration of 5 nM and synchronized by serum depletion followed by growth in media for 12 hours. Then, the cells were treated with BrdU for 30 minutes before being harvested for analysis. (**F**) **&** (**G**) Transient overexpression of Rnd3 in A549 cells down-regulated NICD and Hes1 in a dosage dependent manner. Data represent means ± S.D. *p<0.05 compared to control (group 0); #*p*<0.05 compared to group 1; $*p*<0.05 compared to group 3. Data represent means ± S.D.

H358 cell line was derived from lung bronchioalveolar carcinoma and H520 cell line was derived from lung squamouse cell carcinoma. However, the major subtype of NSCLC was lung adenocarcinoma. So we confirmed our study in one lung adenocarcinoma cells, A549, as shown in Fig. 6. Rnd3 was down-regulated along with up-regulated Rho Kinase and Notch1 signaling in A549 cells compared to HBEC cells (Fig. 6A and 6B). We quantified the up-regulation fold in change in two signaling. The results were showed in Fig. 6C. Rho Kinase signaling were up-regulated by almost 4 fold evidenced by the pMYPT1 level; the Notch1 signaling were up-regulated by 5 fold evidenced by NICD level. Inhibition of Notch1 signaling by Compound E blocked the proliferation of A549 cells (Fig. 6D and 6E). The cytotoxicity of Compound E in A549 cell was also evaluated by Trypan blue staining. As showed in Fig. S6 (picture 9 to 12), the 5 nM Compound E caused no significant death compared to baseline condition in A549 cells. Mechanistically, Rnd3 regulated the cell proliferation through NICD/Hes1 signaling. Overexpression Rnd3 suppressed the NICD and Hes1 expression in a dosage dependent manner in A549 cells (Fig. 6F and 6G). Our findings in A549 cells were consistent with the results from H358 and H520 cells, suggesting the Rnd3-NICD-Hes1 signaling was a generally molecular mechanism to regulate NSCLC cells growth. Together, higher NICD and Hes1 expression and lower Rnd3 expression were observed in H520, H358 and A549 cells compared to HBEC cells. Reintroduction of Rnd3 inhibited NICD and Hes1 expression, which resulted in decreased proliferation rates of H520 and H358 cells. Blockage of Notch1 signaling inhibited the cell proliferation in H358, H520 and A549 cells. Rnd3 regulated the NICD abundance through prompting its proteasomal degradation in NSCLCs.

## Discussion

Rnd3 belongs to the Ras superfamily and is a small Rho GTPase that regulates a variety of cell functions and diseases. It was originally identified as a ROCK1 endogenous inhibitor. Rnd3 binds to ROCK1 to inhibit downstream signaling [5]. Recently, studies have emphasized the critical role of Rnd3 in different types of cancer. Rnd3 is down-regulated in many cancers, such as hepatocellular carcinoma [10], [23], mesenchymal tumor cells [12], and prostate cancer [12]. However, in some cancers, Rnd3 is up-regulated, such as in pancreatic tumors [24], [25], colon cancer cell lines [25] and some melanomas [26]. These differences suggest that Rnd3 has specific roles in different cell lineages. Interestingly, as a gene highly related to tumors, research on the role of Rnd3 in NSCLC is extremely deficient. Two studies showed that Rnd3 over-expression may be associated with an unfavorable prognosis in NSCLC patients [27], [28] while the precise role of Rnd3 in NSCLC has not yet been investigated. Here, we reported that Rnd3 is down-regulated in H358, H520 and A549 cells, and this down-regulation promotes the growth of these two cell lines and is dependent upon up-regulated Notch signaling.

One of the most important strategies for curing cancer is inhibition of the uncontrolled tumor cell proliferation. However, a lack of specific inhibition is the major problem faced in the clinic. Understanding the unique regulatory mechanism of the cell cycle in cancer cells would favor the discovery of new drugs and new treatments for cancer. Rnd3 overexpression in fibroblasts inhibits S phase entry by blocking cyclin D1 expression at the post-transcriptional level [13]. In the same study, overexpression of cyclin D1 did not rescue Rnd3-mediated cell cycle arrest, suggesting that other players are involved cell cycle regulation. In prostate cancer cells, Rnd3 expression induces the cells arrested in G2/M rather than in G1, as seen in fibroblasts, suggesting that Rnd3 has the ability to regulate cell cycle progression at different phases [29]. Our data demonstrated that Notch/Hes1 signaling is critical in the Rnd3 deficiency-mediated growth of H358, H520 and A549 cells. This finding broadens the understanding of Rnd3’s function in cancer cell cycle regulation and also provides a potential target for NSCLC treatment.

Rnd3 expression is correlated with cell migration in development and diseases. Two recent studies found that Rnd3 regulated neuron migration through Rho Kinase dependent signaling [30], [31]. Meanwhile in cancers, down-regulation of Rnd3 is closely associated with invasion and metastasis [10], [23], [32]. The potential mechanism may also be through RhoA/ROCK1 signaling. In this study, we showed that both NICD and Rho Kinase are activated in H358, H520 and A549 cells compared to HBEC. Reintroduction of Rnd3 inhibited both signaling pathways. However, only Notch signaling inhibition prevents the hyper-growth of H358 and H520 cells; Rho Kinase inhibition does not. Further studies to investigate the regulation of tumorigenesis, migration and tumor cell apoptosis in response to Rho Kinase inhibition would be necessary and interesting.

Notch is an evolutionarily conserved transmembrane receptor involved in development and diseases. Upon ligand stimulation, Notch can be cleaved by γ-secretase. The intracellular part (NICD) can translocate into the nucleus to initiate transcription. Forced expression of Notch 1 inhibited the growth of A549 cells in culture [2]. However, an independent study has challenged this notion [33]. More recently, a Notch 1 activation mutant has been found in approximately 10% of NSCLC, and the mutations conferred a worse prognosis in patients [4]. This controversy suggests, at least, that 1) function of Notch 1 in NSCLC is diversified in different cells and different cancer stages; 2) Notch1 expression should be considered while treating patients; 3) we urgently need to understand the role of Notch1 in NSCLC. In this study, our data indicated that Notch signaling is activated in H358 and H520 cells, and this activation drove the proliferation of these two cells through Hes1. Rnd3 could repress H358, H520 and A549 cells proliferation by antagonizing Notch signaling in a post-translational manner.

A recently published study highlighted that Rnd3 was a downstream mediator of Notch signaling in squamous cell carcinomas (SCC) in skin epithelia [34]. Those authors suggested that Rnd3 is a transcriptional target of activated Notch1, and Rnd3 depletion suppresses Notch signaling by mediating nuclear translocation of the NICD through an interaction with importins. In our study, we found that Rnd3 overexpression suppressed Notch signaling by blocking NICD degradation. These discrepancies may be because 1) a different cell line was used for the study; 2) more likely, these two types of regulation co-exist in cells, working as a feedback mechanism to precisely control Notch signaling.

Expression of Rnd3 express was precisely regulated in human tumor tissues. Rnd3 was up-regulated in colorectal cancer compared to normal human colorectal tissue [35]. While down-regulation of Rnd3 was observation in human liver tumor tissue and human esophageal squamous cell carcinoma tissues compared to the adjacent normal tissues respectively [11], [36]. Although the different expression pattern of Rnd3 was found in different tumors in human, Rnd3 expression was closely associated with tumor cell proliferation, cell migration/metastasis and diagnosis/prognosis. Given the fact that Rnd3 was down-regulated in NSCLC cells and down-regulation of Rnd3 promoted the NSCLC cells proliferation, it would be interesting and necessary to evaluate the Rnd3 expression in human lung cancer tissue specimens.

Taken together, we are the first group to report that Rnd3 is down-regulated in H358, H520 and A549 cells, resulting in a hyper-activation of Notch and Rho Kinase signaling. Mechanistically, Rnd3 regulates the proliferation of H358, H520 and A549 cells through the NICD/Hes1 signaling axis.

## Supporting Information

Figure S1 **Expression of PDK1, a Rho Kinase activator, remains unchanged in H358 and H520 cells compared to HBEC cells.** PKD1 could compete with Rnd3 to bind to ROCK1, activating Rho Kinase signaling. **(A)** The protein expression level of PDK1 does not change in two cancer cell lines compared to HBEC cells. **(B)** Quantification of PDK1 expression suggested no statistical significance in PDK1 expression among the three cells. Data represent means ± S.D.
(TIF)

Figure S2 **Quantification of the ratio of BrdU+ cells to GFP+ cells. (A)** the ratio of BrdU+ cells to GFP+ cells are significant higher in H358 and H520 cells compared to H358-Rnd3 and H520-Rnd3 cells, respectively as shown **(A)** & **(B)**. Data represent means ± S.D.
(TIF)

Figure S3 **Expression of Notch ligands in H358, H520, H358-Rnd3 and H520-Rnd3 cells. (A)** Expression of selected Notch ligands, Jag-1, Jag-2, Dll-1, Dll-4, did not change in H358 cells compare to H358-Rnd3 cells. **(B)** The expression of Notch ligands did not change in H520 cells compared to H520-Rnd3 cells. The data are representive for 3 experiment repeats. Data represent means ± S.D.
(TIF)

Figure S4 **Effect of Rho Kinase inhibitor, Fasudil, on the proliferation rate of H358 and H520 cells.** Treatment of Fasudil did not change the proliferation of H358 and H520 compared to DMSO treatment group. **(A)** H358 cells were treated with Fasudil followed by BrdU incorporation. **(B)** Quantification of BrdU intensity normalized by DAPI. **(C)** H520 cells were treated with Fasudil followed by BrdU incorporation. **(D)** Quantification of BrdU intensity normalized by DAPI. The experiments were repeated 3 times, and images were required under 20x objective. BrdU-positive cells were quantified from 8 images taken from four slides. Data represent means ± S.D.
(TIF)

Figure S5 **Relative mRNA expression level of NICD and Hes1 in response to Rnd3 overexpression.** NICD mRNA remains no change when Rnd3 was over expressed represented by empty bar. The Hes1 mRNA was down-regulated along with Rnd3 overexpression represented by the black filled bar. The mRNA was normalized to GAPDH expression. *p<0.05 compared to control (group 0); **#** *p*<0.05 compared to group 1; **$** *p*<0.05 compared to group 3. Data represent means ± S.D.
(TIF)

Figure S6 **Cell viability was evaluated by Trypan Blue staining in response to Compound E.** No cell death was detected in baseline condition and 5 nM compound E treatment in all three cell lines (picture 1, 5, 9 compared to 2, 6, and 10). Minimal (1%–4%) cell death was observed in response to 50 nM Compound E treatment in three cell lines (picture 3, 7, 11). Massive cell death (>50%) was observed in all three cell lines treated by 500 nM compound E (picture 4, 8, 12). White arrow indicates the dead cells.
(TIF)
