# Supplementary figures and images for: Rnd3 Regulates Lung Cancer Cell Proliferation through Notch Signaling
Source: PLoS One. 2014 Nov 5;9(11):e111897. doi: 10.1371/journal.pone.0111897 (PMC4221162; doi:10.1371/journal.pone.0111897)

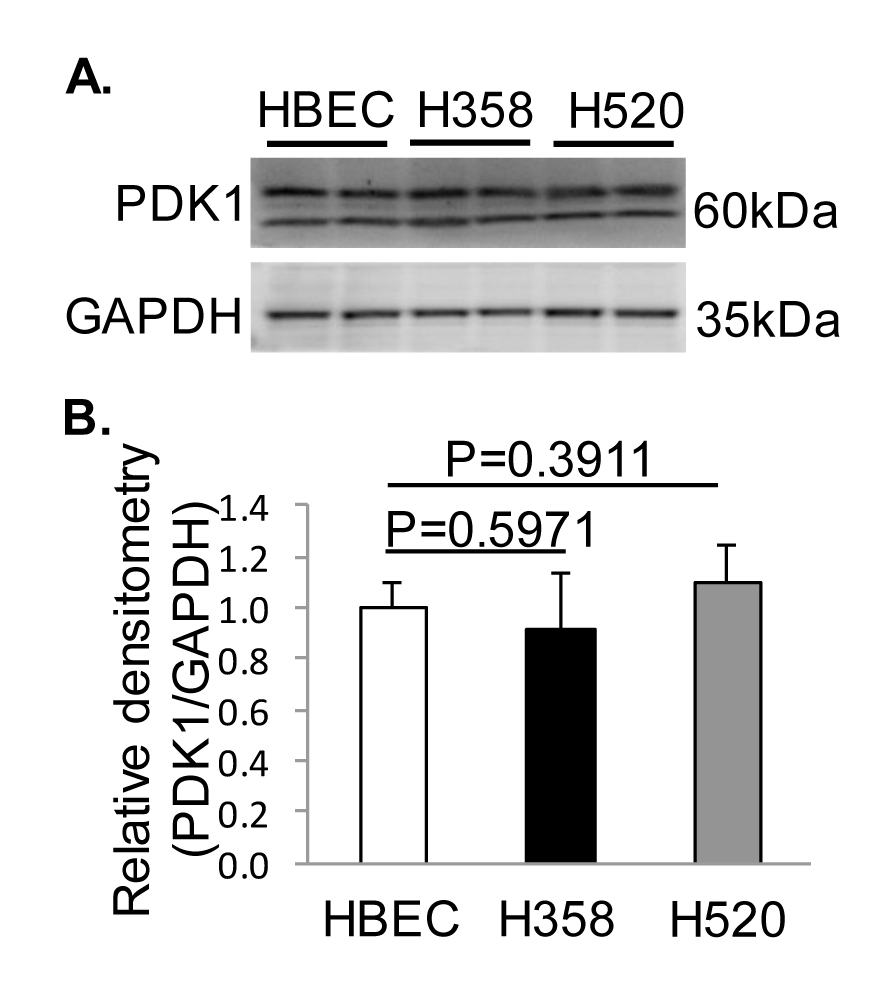

Supplement: Figure S1 — Expression of PDK1, a Rho Kinase activator, remains unchanged in H358 and H520 cells compared to HBEC cells. PKD1 could compete with Rnd3 to bind to ROCK1, activating Rho Kinase signaling. (A) The protein expression level of PDK1 does not change in two cancer cell lines compared to HBEC cells. (B) Quantification of PDK1 expression suggested no statistical significance in PDK1 expression among the three cells. Data represent means ± S.D. (TIF) [file pone.0111897.s001.tif]

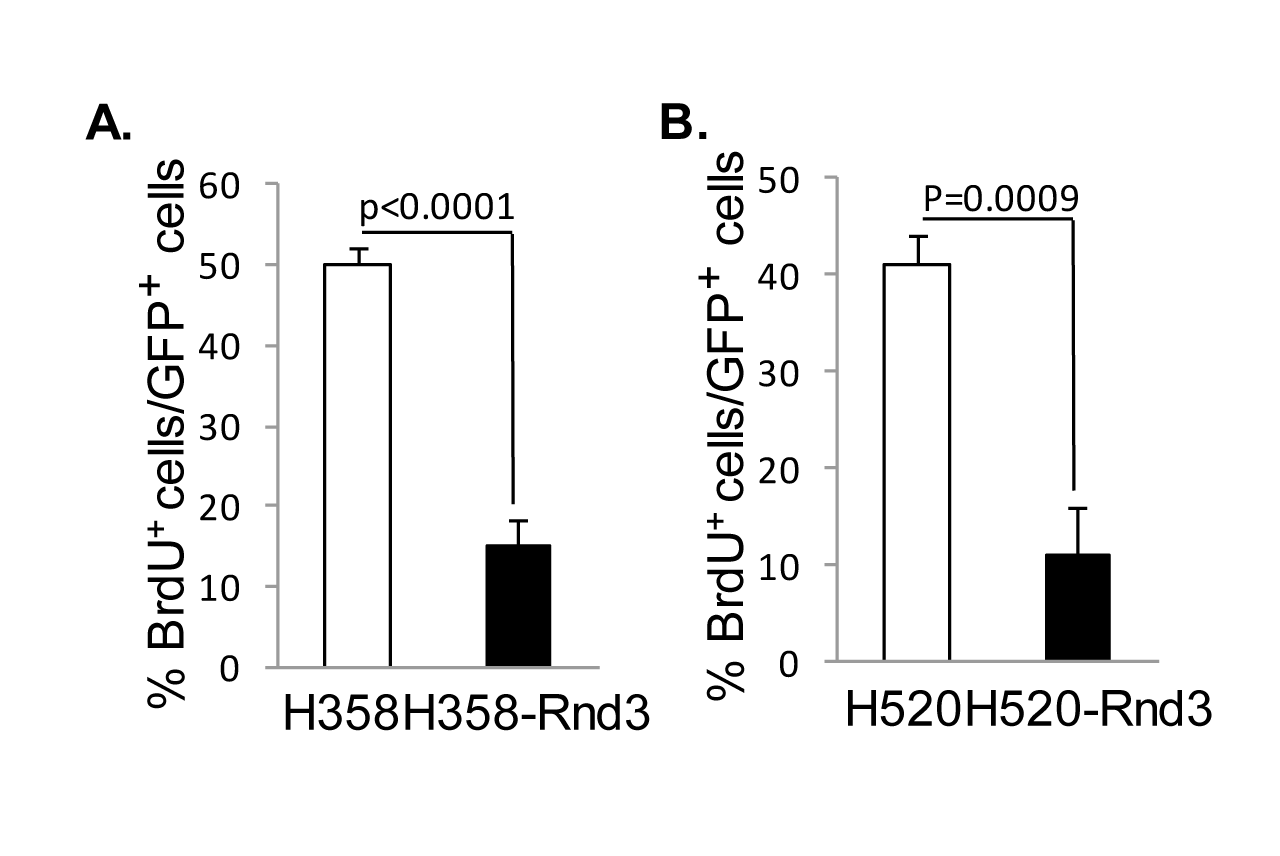

Supplement: Figure S2 — Quantification of the ratio of BrdU+ cells to GFP+ cells. (A) the ratio of BrdU+ cells to GFP+ cells are significant higher in H358 and H520 cells compared to H358-Rnd3 and H520-Rnd3 cells, respectively as shown (A) & (B). Data represent means ± S.D. (TIF) [file pone.0111897.s002.tif]

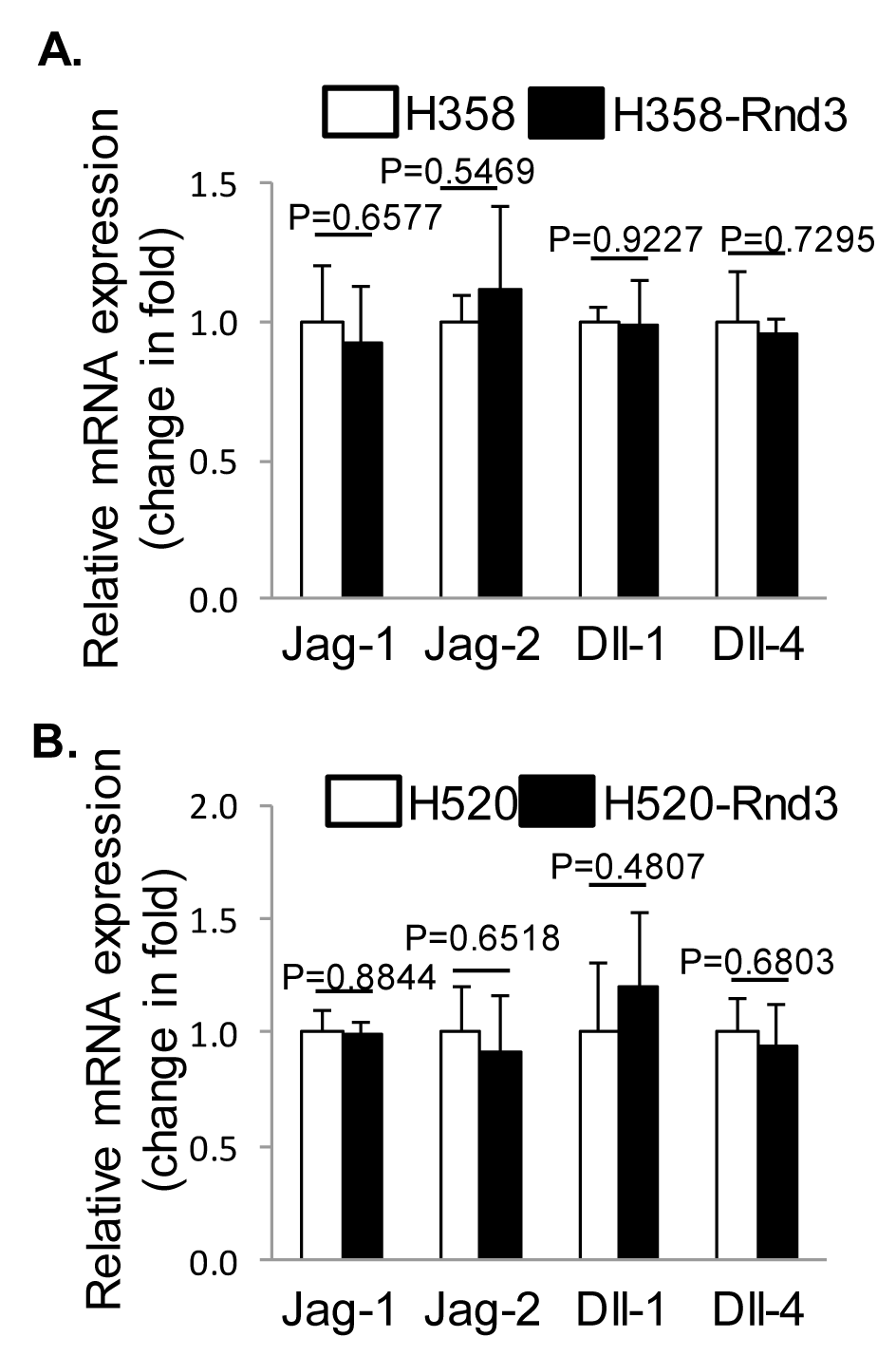

Supplement: Figure S3 — Expression of Notch ligands in H358, H520, H358-Rnd3 and H520-Rnd3 cells. (A) Expression of selected Notch ligands, Jag-1, Jag-2, Dll-1, Dll-4, did not change in H358 cells compare to H358-Rnd3 cells. (B) The expression of Notch ligands did not change in H520 cells compared to H520-Rnd3 cells. The data are representive for 3 experiment repeats. Data represent means ± S.D. (TIF) [file pone.0111897.s003.tif]

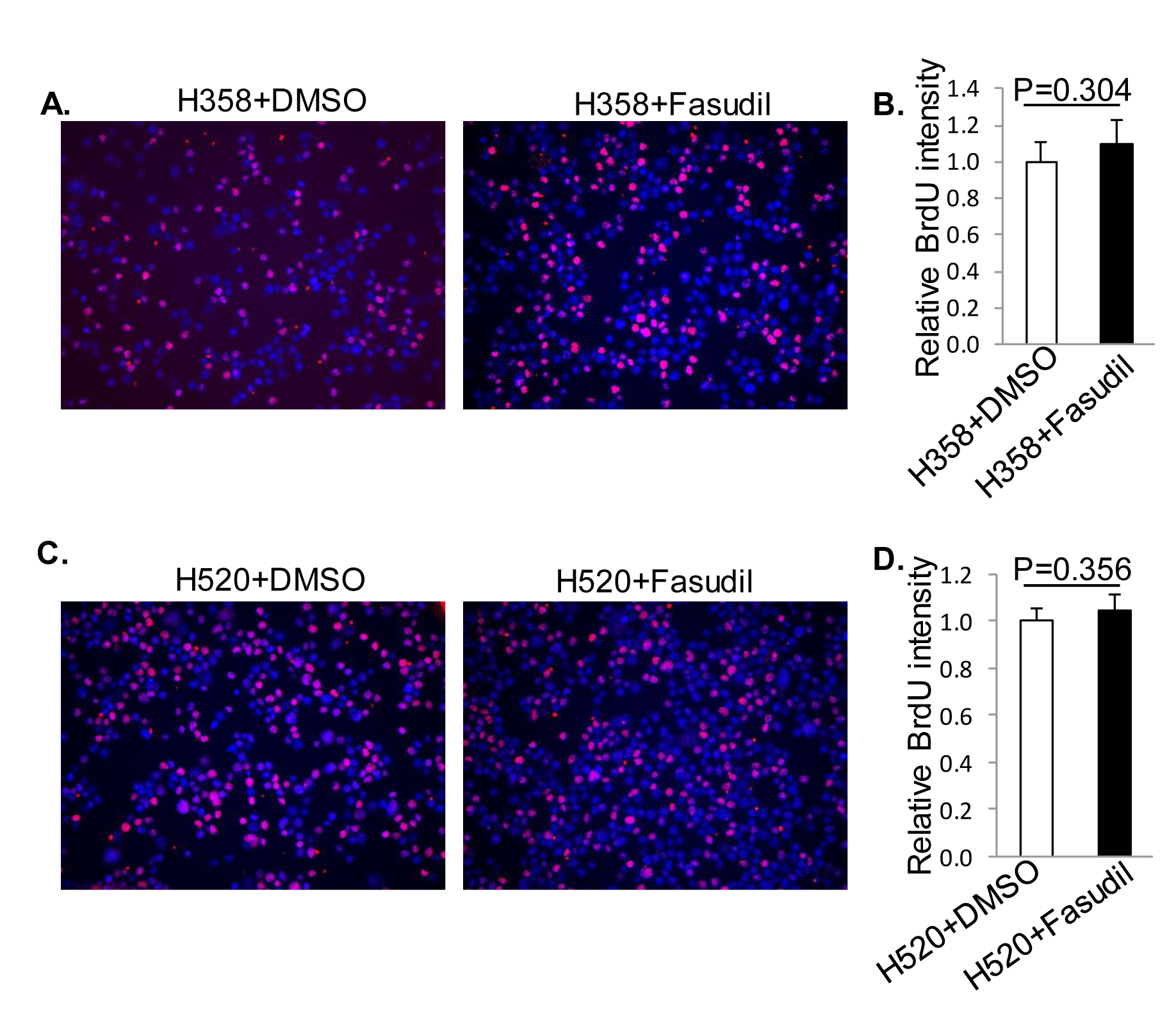

Supplement: Figure S4 — Effect of Rho Kinase inhibitor, Fasudil, on the proliferation rate of H358 and H520 cells. Treatment of Fasudil did not change the proliferation of H358 and H520 compared to DMSO treatment group. (A) H358 cells were treated with Fasudil followed by BrdU incorporation. (B) Quantification of BrdU intensity normalized by DAPI. (C) H520 cells were treated with Fasudil followed by BrdU incorporation. (D) Quantification of BrdU intensity normalized by DAPI. The experiments were repeated 3 times, and images were required under 20x objective. BrdU-positive cells were quantified from 8 images taken from four slides. Data represent means ± S.D. (TIF) [file pone.0111897.s004.tif]

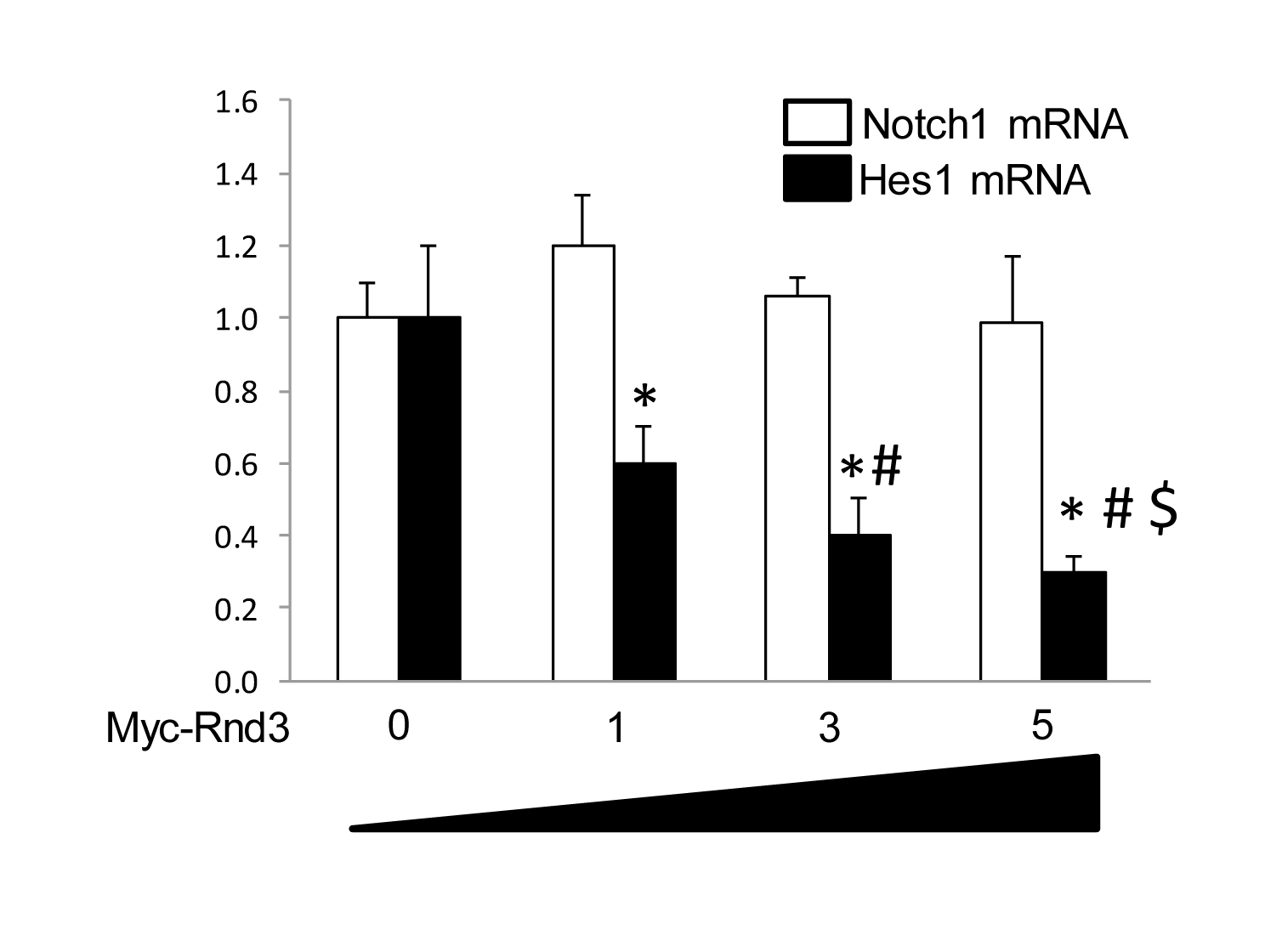

Supplement: Figure S5 — Relative mRNA expression level of NICD and Hes1 in response to Rnd3 overexpression. NICD mRNA remains no change when Rnd3 was over expressed represented by empty bar. The Hes1 mRNA was down-regulated along with Rnd3 overexpression represented by the black filled bar. The mRNA was normalized to GAPDH expression. *p<0.05 compared to control (group 0); # p<0.05 compared to group 1; $ p<0.05 compared to group 3. Data represent means ± S.D. (TIF) [file pone.0111897.s005.tif]

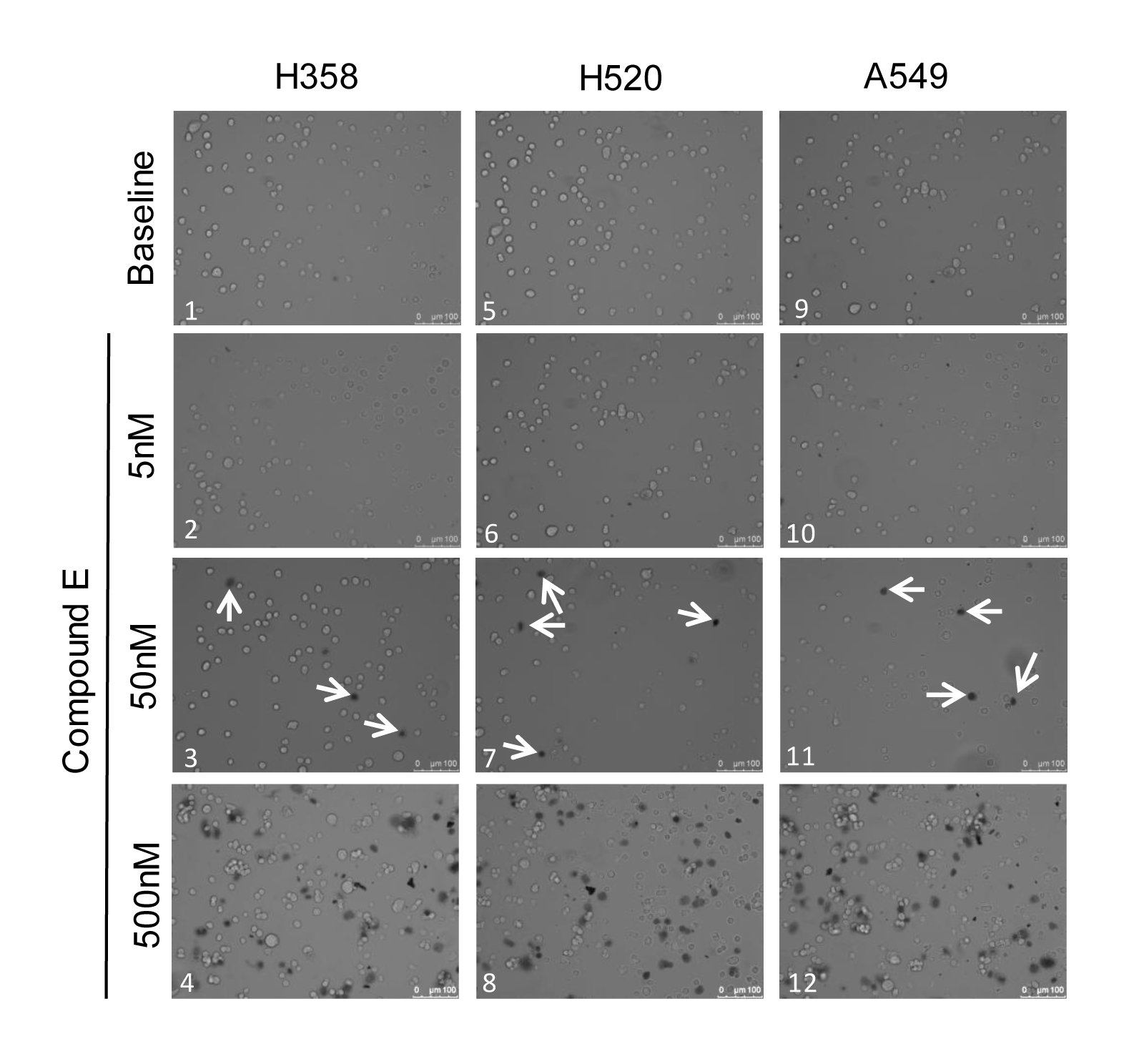

Supplement: Figure S6 — Cell viability was evaluated by Trypan Blue staining in response to Compound E. No cell death was detected in baseline condition and 5 nM compound E treatment in all three cell lines (picture 1, 5, 9 compared to 2, 6, and 10). Minimal (1%–4%) cell death was observed in response to 50 nM Compound E treatment in three cell lines (picture 3, 7, 11). Massive cell death (>50%) was observed in all three cell lines treated by 500 nM compound E (picture 4, 8, 12). White arrow indicates the dead cells. (TIF) [file pone.0111897.s006.tif]
